# The Association between Metabolic Syndrome and Morbid Events in Type 2 Diabetes after a 7-Year Community Management: Beijing Community Diabetes Study 17

**DOI:** 10.1155/2019/5237371

**Published:** 2019-06-12

**Authors:** Guang-Ran Yang, Ming-Xia Yuan, Han-Jing Fu, Gang Wan, Dongmei Li, Timothy D. Dye, Liang-Xiang Zhu, Rong-Rong Xie, Yu-Jie Lv, Jian-Dong Zhang, Xue-Ping Du, Yu-Ling Li, Yu Ji, Yue Li, Xue-Li Cui, Zi-Ming Wang, Shu-Yan Cheng, De-Yuan Liu, Qian Wang, Li Zhou, Ying Gao, Shen-Yuan Yuan

**Affiliations:** ^1^Department of Endocrinology, Beijing Tongren Hospital, Capital Medical University, Beijing, China; ^2^Department of Medical Records and Statistics, Beijing Ditan Hospital, Capital Medical University, Beijing, China; ^3^Clinical and Translational Science Institute, School of Medicine and Dentistry, University of Rochester, Rochester, NY, USA; ^4^Cuigezhuang Community Health Service Center, Beijing, China; ^5^Jinsong Community Health Service Center, Beijing, China; ^6^Yuetan Community Health Service Center of Fuxing Hospital, Capital Medical University, Beijing, China; ^7^Xinjiekou Community Health Service Center, Beijing, China; ^8^Department of Endocrinology, Beijing Aerospace General Hospital, Beijing, China; ^9^Aerospace Central Hospital, Beijing, China; ^10^Sanlitun Community Health Service Center, Beijing, China; ^11^Jiangtai Community Health Service Center, Beijing, China; ^12^Balizhuang Community Health Service Center, Beijing, China; ^13^Zuojiazhuang Community Health Service Center, Beijing, China; ^14^Majiapu Community Health Service Center, Beijing, China; ^15^School Hospital of Central University for Nationalities, Beijing, China; ^16^The First People's Hospital of Dongcheng District, Beijing, China

## Abstract

**Background:**

To examine the association between morbid events and metabolic syndrome (MS) in patients with type 2 diabetes mellitus (T2DM).

**Methods:**

A prospective, longitudinal, multicenter study was conducted at 13 community health centers associated with Beijing Tongren Hospital. From 2008 to 2015, there have been 3,525 T2DM patients being managed based on the Chinese guideline for T2DM. The morbid events included macrovascular events, diabetic kidney disease, ophthalmologic events, cancer, and all-cause death.

**Results:**

At baseline, there were 2,708 people with MS and 817 without MS. After a seven-year management, there were 351 (12.96%) events in MS people and 74 (9.06%) events in people without MS (*p* = 0.003). The prevalence of macrovascular events (6.06%) was much higher in MS people than in people without MS (3.79%, *p* = 0.013). Cox regression analysis showed an association between MS and morbid events even after adjusting for confounding variables (adjusted hazard ratio = 1.44). MS was also associated with macrovascular events (adjusted hazard ratio = 1.96). The occurrence of morbid events and macrovascular events was increased when the numbers of metabolic abnormalities were 1, 2, 3, and 4 (*p* < 0.001). There was no continuously statistically significant difference in the cumulative prevalence of morbid events between patients with MS and patients without MS during the first five years. However, after six or seven years, the cumulative prevalence of morbid events in patients with MS was continuously significantly higher than that in patients without MS (11.00% vs. 8.20%, 12.96% vs. 9.06%, *p* < 0.05).

**Conclusions:**

T2DM with MS had higher incidence of morbid events, especially cardiovascular events, even after integrated management. The occurrence of morbid and macrovascular events increased as the number of metabolic abnormalities increased. MS was associated with increased risk of morbid events by 44% and macrovascular events by 96%. It would take at least six years to observe the association between MS and morbid events in T2DM.

## 1. Introduction

Metabolic syndrome (MS) consists of many metabolic abnormalities, such as obesity, hyperglycemia, hypertension, and dyslipidemia. MS increases the risk of future cardiovascular disease (CVD) [1]. Compared with patients without MS, patients with MS have higher risk for heart attack or stroke and are more likely to die from them [2]. There are several definitions of MS [3–5]. However, presence of MS, regardless of definition, is a significant predictor for CVD in many different populations, which has been reported by many large-scale clinical trials and meta-analyses [6].

Diabetes itself is a high-risk condition for atherosclerotic CVD [4]. CVD is the leading cause of mortality in type 2 diabetes mellitus (T2DM), and CVD risk is about 2- to 8-fold higher in T2DM than in the general population with similar age, sex, and ethnicity [7–9]. Furthermore, CVD is one of the largest contributors to the direct and indirect costs of diabetes [10]. In diabetic patients, MS further increases the risk of CVD [11, 12]. Now, CVD is the leading cause of death in T2DM patients in China. Despite its high prevalence, information on the prospective association of MS with CVD or microvascular complications in Chinese T2DM patients is currently limited. The purpose of this study (the Beijing Community Diabetes Study (BCDS)) was to evaluate the association between MS and morbid events in patients with T2DM following a seven-year management in Beijing communities.

## 2. Methods

### 2.1. Participants

It was a prospective, longitudinal, multicenter study over a seven-year-long management of communities conducted at thirteen community health centers associated with Beijing Tongren Hospital. We selected thirteen community health centers in Beijing using a multistage random sampling method. From August 2008 to July 2009, we recruited all the T2DM patients, aged 20 to 80 years, who had lived in the community for at least five years [13]. A total of 3,525 T2DM patients were recruited from thirteen community health centers including Cuigezhuang, Jinsong, Yuetan, Ping'an, 711, Mingzu, 721, Sanlitun, Jiangtai, Balizhuang, Chongwen, Zuojiazhuang, and Majiapu. We excluded patients with severe disabilities, hepatic failure, renal failure, or schizophrenia [13].

This study was conducted according to the provisions of the Declaration of Helsinki. We obtained written informed consent from all patients and followed them for seven years. The Ethics Committee of Beijing Tongren Hospital, Capital Medical University, had approved this study [13].

### 2.2. Integrated Care

Details in the design, methods, and population of BCDS have been published previously [13]. The major component of integrated care was a collaborative team including endocrinologists and community general practitioners (GPs). Regular follow-up visits were conducted by physicians to adjust medication (at least four times each year). Metabolic targets were set as follows: hemoglobin A1c (HbA1c) < 7.0%, fasting plasma glucose (FPG) < 7.2 mmol/l, blood pressure (BP) < 130/80 mmHg, and low - density lipoprotein cholesterol (LDL - C) < 2.6 mmol/l according to the Chinese guideline for T2DM [14].

A physical examination and laboratory measurements were performed according to the protocol at baseline and each follow-up visit thereafter. BP was measured twice after each patient had been seated for 10 minutes, and the average value was used for analysis. Body mass index (BMI) was calculated as weight divided by height squared (kg/m^2^). Waist circumference (WC) was examined at the level midway between the lower rib margin and the iliac crest. Neck circumference (NC) was measured with the patient's head erect and eye facing forward, horizontally at the upper margin of the laryngeal prominence (Adam's apple) [13, 15].

FPG and HbA1c measurements were performed quarterly, and the lipid profile biannually. Serum creatinine, urinary albumin excretion rate (UAER), and retina examinations were measured annually. FPG, lipid profiles, creatinine, and uric acid (UA) were measured by an autoanalyzer. HbA1c was measured by using a Bio-Rad Variant hemoglobin analyzer. Eight-hour overnight urine was collected to measure UAER. Nonstereoscopic 45° photographs of the central retina were taken in both eyes (Camera CR-DGi; Canon Inc., Tokyo, Japan). Diabetic retinopathy was graded by fundus photograph by ophthalmologists masked to the patients' clinical characteristics according to the modified Airlie House Classification system. The presence of at least one microaneurysm was used as the minimum criterion for diagnosis of diabetic retinopathy [16, 17]. According to the Chinese guideline for T2DM, medication adjustment strategies were performed [14].

Over a follow-up period of seven years, occurrences of morbid events, including macrovascular events, diabetic kidney disease (DKD) events, ophthalmologic events, cancer, and all-cause death, were collected. Macrovascular events were defined as CVD events (myocardial infarction, coronary revascularization, and admission for heart failure) and stroke (cerebral infarction and hemorrhagic stroke confirmed by cranial computed tomography/magnetic resonance imaging). DKD was defined as newly onset microalbuminuria and aggravation of diabetic nephropathy, including clinical grade proteinuria, a 2-fold increase in serum creatinine, and renal replacement therapy. Ophthalmologic events were defined as photocoagulation and retinal detachment. Morbid events were patient-reported. After patient reporting, the GPs collected the patients' medical records related to the events. The morbid event committee comprising of cardiologists, neurologists, and endocrinologists then reviewed the records and confirmed the event.

### 2.3. Definition of MS

MS was defined according to the 2009 Joint Statement definition of MS [18]. This definition includes FPG ≥ 5.6 mmol/l and/or known treatment for hyperglycemia, systolic blood pressure (SBP) ≥ 130 mmHg and/or diastolic blood pressure (DBP) ≥ 85 mmHg or known treatment for hypertension, abdominal obesity (WC ≥ 90 cm in men, WC ≥ 80 cm in women), triglyceride (TG) ≥ 1.7 mmol/l or taking medication for elevated TG, high − density lipoprotein cholesterol (HDL − C) ≤ 1.0 mmol/l in men and ≤1.3 mmol/l for women, or taking medication. MS was defined as the coexistence of 3 or more components including hyperglycemia.

### 2.4. Statistical Analysis

EpiData 3.0 software was used to establish the database for our study. All data were analyzed using the statistical analysis software SAS. All the people were categorized into the non-MS group and the MS group according to the clinical characteristics at baseline. Mean (standard deviations (SD)), *n* (percentage), and median (range) were used to describe the characteristics of our study participants. The independent *t*-test was used to assess the difference in groups for continuous variables. The rank-sum test was utilized for nonnormally distributed diabetic duration and UA. The occurring time of a morbid event was defined when the first morbid event occurred. Only the first morbid event was included in the statistical analysis if the patient had more than one morbid event during the seven-year follow-up. The chi-square test was used to compare the occurrence of morbid events occurring between the enrollment and December 2015 in different groups. Kaplan-Meier analysis and log-rank test were used to assess the association between the cumulative occurrence of morbid events and MS. The Cox regression model was used to identify the association between MS and morbid events. Gender, age, education attainment, diabetic duration, the baseline prevalence of related chronic diseases, HbA1c, and UA were adjusted in the multivariate Cox regression model. All adjustments were made for baseline variables. A hazard ratio (HR) with corresponding 95% confidence interval (CI) was reported. The significance level for all statistic tests was set as 0.05.

## 3. Results

### 3.1. Clinical Characteristics at Baseline

A total of 3,525 patients with an average age of 61 ± 10 years were examined. There were 2,708 patients with MS and 817 without MS at baseline. The median duration of diabetes was 3.9 (range 0.1-9.1) years at baseline. In addition to MS components, differences in gender, education attainment, BMI, NC, FPG, postprandial glucose, and UA were also statistically significant between the MS group and the non-MS group (*p* < 0.001, *p* < 0.001, *p* < 0.001, *p* < 0.001, *p* = 0.009, *p* = 0.011, and *p* < 0.001, respectively, Table 1). HbA1c were higher in the MS group (7.3 ± 1.5%, Table 1). At baseline, the prevalence of related chronic diseases including coronary heart disease, stroke, diabetic nephropathy, diabetic retinopathy, and cancer was calculated. The prevalence of related chronic diseases in the non-MS group and the MS group was 26.81% (219/817) and 37.44% (1,014/2,708, *p* < 0.001), respectively. The prevalence of macrovascular disease (coronary heart disease and stroke) was higher in the MS group (30.50%, 826/2,708) than in the non-MS group (20.81%, 170/817, *p* < 0.001).

### 3.2. Clinical Characteristics after the Seven-Year Management

After seven years of management, statistically significant differences were found in BMI, WC, NC, SBP, TG, HDL-C, and UA between the non-MS and MS groups, when all the people were divided into the non-MS and MS groups according to the baseline clinical characteristics (*p* < 0.001, *p* < 0.001, *p* < 0.001, *p* = 0.003, *p* < 0.001, *p* = 0.001, and *p* = 0.040, respectively). FPG, HbA1c, LDL-C, and UA values were higher in the MS group (Table 2).

### 3.3. Morbid Events after the Seven-Year Management

**Figure 1:**
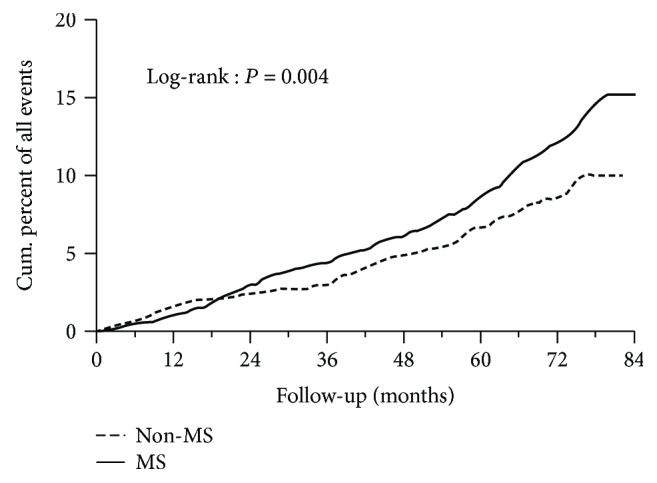
Kaplan-Meier curve with morbid events in people with or without MS during the 7-year management. MS: metabolic syndrome.

After the seven-year management, differences in the occurrence of morbid events were evident between the two groups. There were 351 (12.96%) events in the MS group and 74 (9.06%) events in the non-MS group (*p* = 0.003). The Kaplan-Meier curve and log-rank test also showed that the occurrence in the MS group was higher than that in the non-MS group (*p* = 0.004, Figure 1). The prevalence of macrovascular events was much higher in MS people (6.06%) than in people without MS (3.79%, *p* = 0.013). The prevalence of cardiovascular events was significantly higher in the MS group (3.36%) than in the non-MS group (1.96%, *p* = 0.041, Table 3).

All the people were divided into four groups according to the number of metabolic abnormalities (diabetes, hypertension, dyslipidemia, and abdominal obesity) at baseline. The occurrence of morbid events was increased when the numbers of metabolic abnormalities were 1, 2, 3, and 4 (4.67%, 10.40%, 12.42%, and 13.89%, respectively), which was further confirmed by the Cochran-Armitage trend test (*Z* = −3.15, *p* < 0.001). Similar results were found in macrovascular events. The occurrence of macrovascular events was increased when the numbers of metabolic abnormalities were 1, 2, 3, and 4 (2.34%, 4.24%, 5.61%, and 6.75%, respectively, *Z* = −3.84, *p* < 0.001).

**Figure 2:**
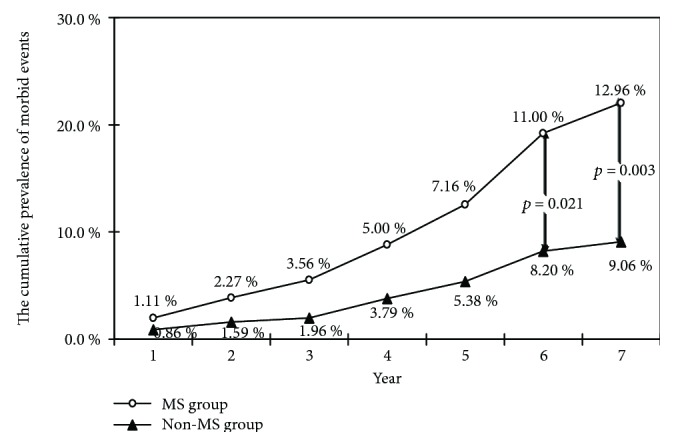
The cumulative prevalence of morbid events in the different groups during the seven-year management. MS: metabolic syndrome.

The cumulative prevalence of morbid events during the seven-year management was compared. There was no continuously statistically significant difference in the cumulative prevalence of morbid events between patients with MS and patients without MS during the first five years of management. However, after six to seven years, the cumulative prevalence of morbid events in patients with MS was significantly higher than that in patients without MS (11.00% vs. 8.20%, 12.96% vs. 9.06%, *p* = 0.021, *p* = 0.003, respectively, Figure 2).

### 3.4. Cox Regression Analysis

Cox regression analysis was performed to evaluate the association between MS and morbid events. There was a significant association between MS and the occurrence of morbid events (crude HR = 1.45, 95% CI 1.12-1.88, *p* = 0.004), even after adjusting for gender, age, HbA1c, duration of diabetes, educational attainment, UA, the use of sulfonylurea and UAER, and the baseline prevalence of related chronic diseases (adjusted HR = 1.44, 95% CI 1.07-1.93, *p* = 0.016). HbA1c, male, age, and lower educational attainment were all risk factors for morbid events. Moreover, MS were also significantly associated with the occurrence of macrovascular events (crude HR = 1.64, 95% CI 1.10-2.43, *p* = 0.015), after adjusting for the same confounding variables (adjusted HR = 1.96, 95% CI 1.19-3.23, *p* = 0.008).

## 4. Discussion

In this study, patients with MS had higher occurrence of morbid events than patients without MS, especially CVD events, even after the seven-year integrated management. Only after six or seven years, the cumulative occurrence of morbid events in patients with MS was significantly higher than that in patients without MS. MS was associated with the occurrence of morbid events, especially macrovascular events, even after adjusting for confounding factors. Furthermore, the occurrence of morbid events and macrovascular events increased as the number of metabolic abnormalities increased.

The incidence of CVD is greatly increased in the setting of T2DM. The risk of various CVD events in middle-aged diabetic patients increased two- to threefold compared with that in patients without diabetes [19, 20]. MS often culminates in T2DM, which carries a particularly high risk for both cardiovascular events and other complications [21, 22]. In our study, the prevalence of MS in T2DM was 76.8%. In other studies referring to T2DM, the prevalence of MS is about 60-75% [23–25]. The prevalence of MS in T2DM, by any definition of the MS, is higher than that in healthy people [26, 27].

It is recognized that both MS and T2DM are risk factors for CVD. T2DM patients with MS may have elevated risk for cardiovascular events. Previous studies have evaluated the effects of MS on CVD in T2DM patients. In a cross-sectional study conducted in Chinese T2DM patients, it was found that T2DM patients with MS have increased cardiovascular risk scores [28]. Metascreen, a cross-sectional survey of MS and clinically detected diabetes complications, performed in 8,497 Italian patients, also reported that either definition of MS was an independent risk factor for all complications including CVD and retinopathy in T2DM [29].

In this study, T2DM patients with MS had higher incidence of morbidity, especially CVD events, even after a seven-year integrated management. In the Verona Diabetes Complications Study, after a mean of 4.5 years' follow-up, the presence of MS was associated with an almost fivefold increase in CVD risk in T2DM patients [30]. A longitudinal ten-year follow-up of the Strong Heart Study cohort had also found that MS was significantly associated with higher incidence of cardiovascular events in the diabetic group [31]. However, in other prospective studies, there were different results. The higher prevalence of MS in diabetes was not associated with the higher risk for cardiovascular events and other complications in some studies [25, 32]. It may be related to different definitions of MS in these studies. There were studies that showed that different definitions of MS had a different association with CVD, even in the same population. An observational cohort study was performed on a consecutive series of 882 Caucasian T2DM outpatients. From the Cox regression analysis with adjustment for sex, age, and its individual components, diagnosis of MS with the National Cholesterol Education Program (NCEP) Adult Treatment Panel III (ATP III) criteria, but not with the International Diabetes Federal (IDF) criteria, was significantly associated with higher mortality [33]. Tong et al. found that the IDF criteria of MS failed to predict coronary heart disease in T2DM patients; in contrast, the NCEP ATP III definition predicted an increased risk of coronary heart disease in patients with T2DM after a median of 7.1 years' follow-up [34]. The Strong Heart Cohort Study, a longitudinal study with a ten-year follow-up, evaluated which definition of MS is a better predictor for CVD events. The results showed that compared to the NCEP ATP III and IDF definitions, MS risk for CVD was the greatest using the WHO definition in the diabetic group [31]. A prospective analysis of a consecutive cohort of 5,202 Chinese T2DM patients also showed that the WHO criterion has a better discriminative power over the NCEP criterion for predicting death in Chinese T2DM patients [35]. Besides the definition of MS, the follow-up period should be considered. In the REACH registry, MS was not associated with an increased risk of cardiovascular events in patients with diabetes [36]. In the REACH study, the follow-up period was four years, which might not be long enough to show the effects of MS on CVD events. In our study, there was no continuously statistically significant difference in the cumulative occurrence of morbid events between patients with MS and patients without MS in the first five years of management. Only after six or seven years, the cumulative occurrence of morbid events in the MS group was significantly higher than that in the non-MS group. It indicates that MS in T2DM patients might be a useful risk assessment tool for estimating an over five-year risk but not for a short-term risk (less than five years) assessment. If the follow-up lasts long enough, the significant association between MS and CVD in T2DM patients might be observed.

Moreover, the occurrence of macrovascular events was increased with the increase in the number of metabolic abnormalities in this study. It was confirmed by other studies. A population of individuals aged 43–84 years was evaluated from 1988 to 1990 and was reevaluated five years later, to determine whether components of MS precede the five-year incidence of CVD. It was shown that the risk of incident CVD five years later increased with the number of the components present [1]. In another cross-sectional study conducted in Chinese T2DM inpatients , the Framingham Risk Score and ten-year cardiovascular risk were increased with the components of MS in T2DM patients with MS [28].

There were limitations in our study. The length of the management (seven years) may not be long enough to measure the effects of MS on morbid event incidence in T2DM patients. In addition, the baseline difference in HbA1c between the two groups may have influenced the association of MS on morbid event incidence, especially microvascular complications, after the seven-year management. Tight glucose control may increase the high risk of morbid events, especially macrovascular events [37]. In this study, the mean age was 61 years. To avoid hypoglycemic events, the HbA1c target was set as below 7.0%. Thirdly, the different definitions of MS may affect the results. We selected the Joint Statement definition for MS proposed by six international major organizations [18], not the Chinese Diabetes Society definition. Further studies are needed to confirm whether different definitions of MS will affect this association between MS and morbid events in Chinese diabetic people.

## 5. Conclusions

In this study, T2DM patients with MS had higher occurrence of morbid events than T2DM patients without MS, especially CVD events, even after integrated management. The occurrence of morbid events and macrovascular events increased as the number of metabolic abnormalities increased. MS was associated with increased risk of morbid events by 44% and macrovascular events by 96%. However, it would take at least six years to observe the association between MS and the occurrence of morbid events in T2DM patients.

## Figures and Tables

**Table 1:** Baseline clinical characteristics in different groups.

Variable	Non-MS group	MS group	*p*
	(*n* = 817)	(*n* = 2,708)	
Age (year)	61 ± 11	62 ± 11	0.336
Gender (*n* (%))			
Male	473 (57.89)	942 (34.79)	<0.001
Female	344 (42.11)	1766 (65.21)	
Educational attainment (*n* (%))			<0.001
Illiteracy or elementary school	102 (12.52)	522 (19.44)	
Middle school	511 (62.70)	1616 (60.19)	
College or academic degree	202 (24.79)	547 (20.37)	
Diabetic duration (year)	4.0 (0.3, 9.3)	3.8 (0.0, 9.1)	0.227
Body mass index (kg/m^2^)	23.16 ± 2.99	27.77 ± 66.35	<0.001
Waist circumference (cm)	81 ± 8	91 ± 9	<0.001
Neck circumference (cm)	35 ± 3	37 ± 4	<0.001
Systolic blood pressure (mmHg)	124 ± 11	131 ± 14	<0.001
Diastolic blood pressure (mmHg)	76 ± 8	78 ± 9	<0.001
Fasting plasma glucose (mmol/l)	7.56 ± 2.49	7.83 ± 2.56	0.009
Postprandial glucose (mmol/l)	10.18 ± 4.00	10.63 ± 5.06	0.011
Hemoglobin A1c (%)	7.1 ± 1.6	7.3 ± 1.5	<0.001
Hemoglobin A1c (mmol/mol)	53.9 ± 17.6	56.5 ± 16.7	<0.001
Triglyceride (mmol/l)	1.16 ± 0.67	2.07 ± 1.47	<0.001
Total cholesterol (mmol/l)	5.09 ± 1.06	5.19 ± 1.20	0.03
High-density lipoprotein cholesterol (mmol/l)	1.50 ± 0.43	1.29 ± 0.47	<0.001
Low-density lipoprotein cholesterol (mmol/l)	2.98 ± 0.93	3.05 ± 0.93	0.049
Uric acid (*μ*mol/l)	260 (209, 324)	289 (232, 348)	<0.001
Urinary albumin excretion rate (*μ*g/min)	6.6 (4.5, 12.2)	8.3 (5.2, 19.0)	<0.001
Smoking (*n* (%))	139 (17.01)	403 (14.88)	0.139
Antihypertension medication (*n* (%))	316 (38.68)	1836 (67.80)	<0.001
Antihyperglycemic medication (*n* (%))	753 (92.17)	2475 (91.40)	0.487
Metformin (*n* (%))	247 (30.23)	694 (25.63)	0.009
Sulfonylurea (*n* (%))	327 (40.02)	1270 (46.90)	0.001
Insulin (*n* (%))	166 (20.32)	595 (21.97)	0.314
Statin (*n* (%))	126 (15.42)	607 (22.42)	<0.001

Values are expressed as means ± SD, numbers (%), or median (range). Diabetic duration, uric acid, and urinary albumin excretion rate: median (range); the nonparametric test was used. MS: metabolic syndrome.

**Table 2:** Clinical characteristics of the different groups after seven years of management.

Clinical measurement	Non-MS group (*n* = 563)	MS group (*n* = 1,836)	*p* value
Body mass index (kg/m^2^)	23.48 ± 2.97	25.78 ± 3.12	<0.001
Waist circumference (cm)	83 ± 8	89 ± 9	<0.001
Neck circumference (cm)	35 ± 3	36 ± 4	<0.001
Systolic blood pressure (mmHg)	124 ± 8	125 ± 8	0.003
Diastolic blood pressure (mmHg)	74 ± 6	74 ± 6	0.793
Fasting plasma glucose (mmol/l)	7.17 ± 1.95	7.31 ± 1.94	0.168
Postprandial glucose (mmol/l)	8.73 ± 1.90	8.89 ± 1.89	0.112
Hemoglobin A1c (%)	6.8 ± 1.1	6.9 ± 1.1	0.084
Hemoglobin A1c (mmol/mol)	50.5 ± 11.6	51.6 ± 12.2	0.084
Triglyceride (mmol/l)	1.30 ± 0.71	1.68 ± 1.26	<0.001
Total cholesterol (mmol/l)	4.55 ± 1.01	4.63 ± 1.07	0.117
High-density lipoprotein cholesterol (mmol/l)	1.51 ± 0.51	1.42 ± 0.55	0.001
Low-density lipoprotein cholesterol (mmol/l)	2.54 ± 0.84	2.60 ± 0.83	0.197
Uric acid (*μ*mol/l)	302 (235, 345)	308 (252, 352)	0.040
Urinary albumin excretion rate (*μ*g/min)	7.4 (5.7, 11.8)	8.2 (5.7, 12.7)	0.108

Values are expressed as means ± SD or median (range). Diabetic duration and uric acid: median (range); the nonparametric test was used. MS: metabolic syndrome.

**Table 3:** Morbid events in the different groups after the seven-year management.

Morbid event	Non-MS group	MS group	*χ* ^2^	*p* value	Hazard ratio	95% confidence interval
Cardiovascular events (*n* (%))	16 (1.96)	91 (3.36)	4.19	0.041	1.90	1.08-3.33
Stroke (*n* (%))	15 (1.84)	73 (2.70)	1.91	0.167	1.39	0.80-2.43
Diabetic kidney disease (*n* (%))	16 (1.96)	84 (3.10)	2.98	0.084	1.56	0.91-2.66
Ophthalmologic events (*n* (%))	12 (1.47)	49 (1.81)	0.43	0.513	1.26	0.67-2.37
Cancer (*n* (%))	10 (1.22)	35 (1.29)	0.02	0.879	1.16	0.56-2.42
All-cause deaths (*n* (%))	5 (0.61)	17 (0.63)	<0.01	0.960	0.99	0.36-2.71
Total (*n* (%))	74 (9.61)	351 (13.28)	9.97	0.002	1.45	1.12-1.88

MS: metabolic syndrome.

## Data Availability

All data generated and analysed during the current study are included in this published article.
